# Burden of Undiagnosed Type 2 Diabetes in Diabetic Kidney Disease: A Japanese Retrospective Cohort Study

**DOI:** 10.3390/jcm9072028

**Published:** 2020-06-28

**Authors:** Hayato Tanabe, Haruka Saito, Noritaka Machii, Akihiro Kudo, Kenichi Tanaka, Koichi Asahi, Junichiro James Kazama, Michio Shimabukuro

**Affiliations:** 1 Department of Diabetes, Endocrinology and Metabolism, Fukushima Medical University, Fukushima 960-1295, Japan; htanabe@fmu.ac.jp (H.T.); saito-h@fmu.ac.jp (H.S.); noritaka@fmu.ac.jp (N.M.); a-kudoh@fmu.ac.jp (A.K.); 2 Department of Internal Medicine, Ohara General Hospital, Fukushima 960-8611, Japan; 3 Department of Nephrology and Hypertension, Fukushima Medical University, Fukushima 960-1295, Japan; kennichi@fmu.ac.jp (K.T.); jjkaz@fmu.ac.jp (J.J.K.); 4 Devision of Nephrology and Hypertension, Iwate Medical University, Morioka 020-8505, Japan; asahik@iwate-med.ac.jp

**Keywords:** diabetic kidney disease, undiagnosed diabetes, proteinuria, glomerular filtration rate, type 2 diabetes

## Abstract

The risk of developing diabetic kidney disease (DKD) in patients with undiagnosed diabetes mellitus (UD) has never been evaluated. We studied the burden of UD on the risk of developing DKD in the Japanese population in a single-center retrospective cohort study. The patients with type 2 diabetes mellitus, but without DKD (estimated glomerular filtration rate (eGFR) < 60 mL/min/1.73 m^2^ or proteinuria), were recruited from January 2018 to January 2019; medical records were scrutinized retrospectively from January 2003 until May 2019. The individuals, with diabetes that could not be denied based on past and current records, comprised the undiagnosed diabetes (UD) group whereas those with confirmed diagnosis comprised the diagnosed diabetes (DD) group. The group differences were tested using a Kaplan–Meier curve and Cox proportional hazards model. Among the 408 participants, 164 (40.2%) and 244 (59.8%) comprised the DD and UD groups, respectively. The baseline parameters, including age, male gender, and BMI were comparable between the groups, but the plasma glucose, HbA1c levels, and diabetic retinopathy prevalence were higher in the UD group. The risk of developing DKD (log rank test, *p* < 0.001), an eGFR of < 60 mL/min/1.73 m^2^ (*p* = 0.001) and proteinuria (*p* = 0.007) were also higher in the UD group. The unadjusted and adjusted hazard ratios for DKD were 1.760 ((95% CI: 1.323–2.341), *p* < 0.001) and 1.566 ((95% CI: 1.159–2.115), *p* = 0.003), respectively, for the UD group. In conclusion, this is the first report showing that UD is a strong risk factor for DKD. The notion that a longer duration of untreated diabetes mellitus is involved strongly in the risk of developing DKD warrants the need for the identification and monitoring of UD patients.

## 1. Introduction

The patients who meet the diagnostic criteria, but have not yet been diagnosed for diabetes mellitus, are defined as undiagnosed diabetes mellitus (UD). The definition for UD and its prevalence have been widely discussed [1,2,3,4,5,6,7,8]. Patients with UD may face severer health outcomes as compared to those with diagnosed diabetes (DD). In fact, higher rates of morbidity and mortality have been reported among UD patients in general hospital wards [9] as well as cardiovascular wards [10,11,12,13,14].

Type 2 diabetes mellitus is a major risk factor for chronic kidney disease (CKD), and is the leading cause of end-stage kidney disease (ESKD) worldwide [15]. CKD associated with diabetes mellitus is known as diabetic kidney disease (DKD) [16,17,18]. Among the various risk factors for the development and progression of DKD, the duration of diabetes is a common major risk factor [19,20,21,22]. In order to calculate the duration of diabetes, the estimation of the onset of diabetes should be as correct as possible. However, the duration between the onset and diagnosis of diabetes is totally unknown in UD. Therefore, it is presumed that patients with UD may be at a high risk of developing DKD. However, such a notion has never been evaluated.

We evaluated the impact of UD on the risk of developing DKD in the Japanese population in a single-center retrospective cohort study.

## 2. Research Design and Methods

### 2.1. Study Design and Population

This is an observational retrospective cohort study. The study protocol was approved by the Fukushima Medical University Ethics Committee (Number 29118). Written informed consent was obtained from the outpatients recruited between January 2018 and January 2019 in Department of Diabetes, Endocrinology and Metabolism, School of Medicine, Fukushima Medical University Hospital. This study was conducted according also to the Ethical Guidelines for Medical and Health Research Involving Human Subjects enacted by MHLW of Japan (http://www.mhlw.go.jp/file/06-Seisakujouhou-10600000-Daijinkanboukouseikagakuka/0000069410.pdf and http://www.mhlw.go.jp/file/06-Seisakujouhou-10600000-Daijinkanboukouseikagakuka/0000080278.pdf). Among the 645 patients who gave written informed consent, 144 patients who were either non-diabetic or had type 1 diabetes mellitus were excluded from the study. The remaining 501 patients with type 2 diabetes mellitus were enrolled in the present study and their paper and/or electrical medical records were obtained. Their first visit to our hospital, ranged from January 2003 until January 2019, was considered as baseline and medical records were obtained until May 2019. The parameters such as age, male gender, history of diabetes, family and social history, medical checkup history, complications, medications, laboratory data, and all dates were recorded. The laboratory parameters such as hemoglobin A1c (HbA1c), estimated glomerular filtration rate (eGFR), and qualitative proteinuria by urine stick test were also recorded. Seventy-one patients who were diagnosed with DKD at the baseline and twenty-two patients with non-diabetic kidney diseases (chronic glomerulonephritis, vasculitis, polycystic kidney disease, and renal cancer) were excluded from the analysis; therefore, 408 patients out of 501 were finally included in the analysis.

### 2.2. Definition

Diabetes mellitus was described when a patient had fasting plasma glucose ≥ 126 mg/dL, random plasma glucose ≥ 200 mg/dL, and/or HbA1c ≥ 6.5% (48 mmol/mol), or use of anti-diabetic medications during the first visit at our hospital or during prior medical checkup at any medical institution [23]. In order to strictly determine the presence of diabetes mellitus, we did not consider self-reported diabetes, but collected the data that could be objectively confirmed. The persons whose presence of diabetes could not be confirmed, based on the past objective records, were categorized into the undiagnosed diabetes (UD) group, while the others with confirmed diagnosis were categorized into the diagnosed diabetes (DD) group. The definition of DKD was based on an eGFR < 60 mL/min/1.73 m^2^ or proteinuria 1+ with dipstick urine test. The primary endpoint of this study was the onset of DKD. We calculated the eGFR by using the Japanese formula for GFR estimation, i.e., eGFR (mL/min/1.73 m^2^) = 194 × serum creatinine (mg/dL)^−1.094^ × age (years)^−0.287^ [24]. Hypertension was defined as systolic blood pressure ≥ 140 mmHg or diastolic blood pressure ≥ 90 mmHg or taking antihypertensive drugs. Dyslipidemia was defined as total cholesterol ≥ 220 mg/dL or triglyceride ≥ 150 mg/dL or HDL cholesterol < 40 mg/dL or LDL cholesterol ≥ 140 mg/dL or taking antihyperlipidemic drugs. The definition of macroangiopathy was coronary artery disease or stroke or peripheral artery disease.

### 2.3. Statistical Analysis

The continuous and parametric values are expressed as mean ± standard deviation, and the nonparametric variables as median (first quartile-third quartile). The two-tailed unpaired Student′s *t*-test and Mann–Whitney U test were used for parametric and non-parametric data, respectively. The categorical variables are shown as a percentage and were analyzed using the Chi-square test. The cumulative incidence was done with the Kaplan–Meier curve and differences between groups were analyzed by log rank test. The date of the index measurements of the eGFR and proteinuria at baseline was regarded as time zero on the Kaplan–Meier curves for the development of DKD, eGFR < 60 or proteinuria and the patients were censored when they had developed the incidence of renal target events or dropped from regular visits to our hospital. The Cox proportional hazards model was used to determine the independent contributions of UD to the development of DKD, a decline in eGFR (< 60 mL/min/1.73 m^2^) or proteinuria after adjusting for age, male gender, BMI, HbA1c (at baseline and follow-up), baseline eGFR, current or past smoking, and comorbidities (hypertension, dyslipidemia, macroangiopathy, and diabetic retinopathy). The values of *p* < 0.05 were considered as statistically significant. The statistical analyses were conducted using SPSS version 25 (SPSS, Inc., Chicago, IL, USA).

## 3. Results

### 3.1. General Characteristics

The baseline characteristics of the study participants are shown in Table 1. Among the 408 participants with type 2 diabetes mellitus, 164 (40.2%) were with previous DD and 244 (59.8%) were with previous UD. No significant differences with respect to male gender, BMI, current or past smoking, family history, hypertension, dyslipidemia, and macroangiopathy were observed between the two groups. However, besides the levels of random plasma glucose and HbA1c, the prevalence of diabetic retinopathy was also higher in the UD patients. No significant difference in the baseline eGFR was observed between the two groups.

### 3.2. Univariate and Multivariate Analysis

**Figure 1:**
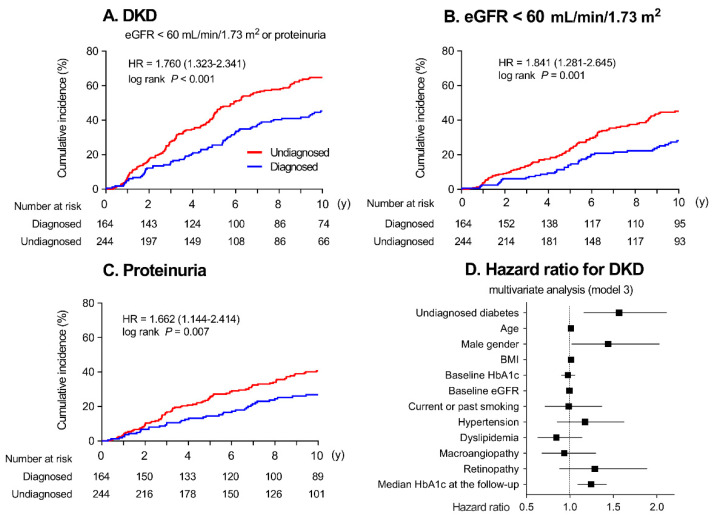
Kaplan–Meier curves for the development of (**A**) diabetic kidney disease (DKD: eGFR < 60 mL/min/1.73 m^2^ or proteinuria), (**B**) eGFR < 60 mL/min/1.73 m^2^, and (**C**) proteinuria in patients with diagnosed (blue lines) or undiagnosed (red lines) diabetes mellitus. (**D**) Hazard ratios for developing DKD in undiagnosed diabetes (UD) patients (Cox proportional hazards model 3)

The results of Kaplan–Meier survival analysis are shown in Figure 1. The median observation time was 6.0 (2.0–10.0) years. A total of 217 patients developed DKD during the observation time, 116 (53.9%) patients developed DKD with eGFR < 60 mL/min/1.73 m^2^ and 101 (46.5%) with proteinuria. The risk of developing DKD was higher in the UD patients than in the DD patients (Figure 1A). The development of eGFR < 60 mL/min/1.73 m^2^ (Figure 1B) and proteinuria (Figure 1C) were also higher in the UD patients. As shown in Table 2, UD was a significant variable for the onset of DKD (hazard ratio (HR): 1.760, 95% CI: 1.323–2.341, *p* < 0.001). Among the other independent variables, male gender, baseline eGFR, diabetic retinopathy, and median HbA1c at follow-up were significant risk variables for the onset of DKD.

In the Cox proportional hazards model, adjusted for age, male gender, BMI, baseline HbA1c, and baseline eGFR (model 1), UD was a significant variable for the onset of DKD (adjusted HR: 1.706, 95% CI: 1.271–2.290, *p* < 0.001). In model 2, which was adjusted for current or past smoking, hypertension, dyslipidemia, macroangiopathy, and any diabetic retinopathy, UD again was a significant variable for the onset of DKD (adjusted HR: 1.647, 95% CI: 1.223–2.219, *p* = 0.001). In model 3, UD was still significant after adjustment for HbA1c levels at follow-up. A forest plot of model 3 is shown in Figure 1D.

**Figure 2:**
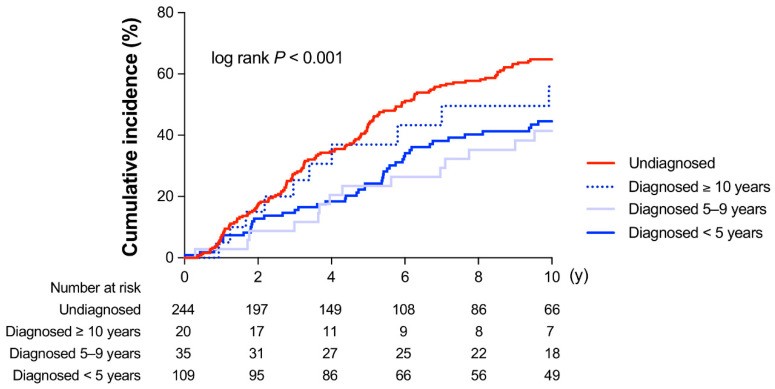
Kaplan–Meier curves for the development of diabetic kidney disease (DKD: eGFR < 60 mL/min/1.73 m^2^ or proteinuria) in patients with diagnosed (blue lines) or undiagnosed (red line) diabetes mellitus. The diagnosed patients were categorized accordingly: Diagnosed < 5 years, Diagnosed 5–9 years, Diagnosed ≥ 10 years duration.

To evaluate the effects of baseline duration of diabetes and the incidence of DKD, we performed Kaplan–Meier survival analysis against the categories UD, DD < 5 years, DD 5–9 years, DD ≥ 10 years duration (Figure 2). The risk of developing DKD was larger in DD ≥ 10 years than in DD < 5 years and DD 5–9 years.

## 4. Discussion

The present study evaluated UD as a risk factor for the onset of DKD in a Japanese single-center retrospective cohort study. We concluded two major points. Firstly, UD was a strong and independent risk factor for developing DKD, even when adjusted for known DKD risks such as current or past smoking, hypertension, dyslipidemia, macroangiopathy, diabetic retinopathy, and median HbA1c at follow-up. Secondly, UD was a significant risk factor for both the decline in eGFR and the onset of proteinuria. To the best of our knowledge, this is the first study showing that UD is a risk factor for developing DKD. The notion that the longer duration of untreated diabetes mellitus may be the risk factor for DKD onset among UD patients warranted us to validate it scientifically.

### 4.1. UD and the Risk of Developing DKD

The current study found that the risk of developing DKD was higher in UD patients as compared to those with DD. A few reports in the literature have examined the relationship between UD and DKD. In a cross-sectional study using the data from National Health and Nutrition Survey (NHANES), Plantinga et al. reported a tendency to a high prevalence of CKD in UD patients. The risk factor for each condition was in the sequence: No diabetes < pre-diabetes < DD < UD [25]. Their result is consistent with our findings. On the contrary, Herrington et al. (2018) reported that renal mortality was higher in the DD patients than in the UD patients in the Mexico City Prospective Study conducted between 1998 and 2004 [26]. The Atherosclerosis Risk in Communities (ARIC) study (2018) also showed that the risk of CKD was higher in the DD patients than in the UD patients. The risk factor for each condition was in the sequence: No diabetes < unconfirmed UD < confirmed UD < DD [27]. The findings of the study by Herrington et al. and the ARIC study are contradictory to our findings. The discrepancy among the studies may be explained by two potential reasons. Firstly, there may be a critical difference in the accuracy of diabetes diagnosis. In the two epidemiological studies, DD was defined by self-reported diabetes and/or a single blood test. In contrast, we diagnosed DD not on a self-reported basis, but with two or more blood tests. The accuracy of the diagnosis can be different based on a single blood test as compared to two or more blood tests [23]. Secondly, there may be a difference in the accuracy of estimating diabetes duration. Two or more blood tests at a year’s interval in our study could determine the duration of diabetes more correctly than previous studies could, which did not repeat blood tests at a year’s interval. The most plausible reason for pathophysiological differences between UD and DD is the duration of diabetes [1,2,3,4,5,6,7,8]. We tried to determine the diagnosis of DD and UD based on repeated blood tests, rather than self-reporting, which is suggestive of more accurate diabetes duration estimation as compared to the previous reports.

### 4.2. Potential Mechanisms

Our study, for the first time, found that the reduction in eGFR and the presence of proteinuria, the two components of DKD, were independently related to UD. We discuss below how UD can be related to the decline in eGFR and the onset of proteinuria.

A reduction in eGFR is strongly related to the duration of diabetes [19,28], which can largely differ between UD and DD patients. The duration of diabetes should be defined as the time elapsed since the onset of diabetes, but not since the incidental hospital diagnosis of diabetes [29]. Although it is not possible to estimate the exact duration of UD, one can expect that it may be longer than that of DD. The results of our study suggest that the patients with UD had a longer duration of hyperglycemia as compared to the DD patients. The risk of developing DKD in DD ≥ 10 years was larger than in the other two categories (Figure 2) and the Kaplan–Meier curve was similar to that in UD, suggesting that the longer duration of diabetes may facilitate DKD in UD as in DD ≥ 10 years.

The baseline HbA1c was higher in the UD group, indicating that the UD patients were unprotected from hyperglycemia until start of appropriate diabetes therapy for an undiagnosed period. In addition, the UD patients had a higher prevalence of diabetic retinopathy, which is also suggestive of a longer duration of hyperglycemia [30,31]. Taken together, although previous epidemiological studies [26,27] did not accurately estimate the duration of diabetes, the differences in diabetes duration between UD and DD patients can be critical for the evaluation of eGFR reduction.

The UD patients in our study also showed an increased concentration of urinary protein. The duration of diabetes has been reported to be a risk factor for albuminuria [28,32,33]. If we assume that UD has a longer duration of diabetes compared to DD, the findings of our study are consistent with this observation. Large-scale clinical studies have reported that long exposure to hyperglycemia is strongly linked to DKD [34,35], but various risk factors other than hyperglycemia are also involved in the onset of DKD [19,20,21]. In the current study, UD was associated with the risk of DKD even after adjusting for known risk factors, thereby suggesting that the potential duration of diabetes in UD patients is independently involved as a risk factor for the development of DKD. However, there is an argument about the underlying etiology and the course of development and progression of DKD, including rapidly decreasing eGFR and rapidly increasing proteinuria [16,17,18]. Thus, it may be suggested that the impact of UD on the reduction of eGFR and increase in proteinuria be evaluated separately in future studies.

### 4.3. Study Limitations

Our study has certain limitations. Firstly, the patients were recruited at a university hospital. Patients who have been referred to a university hospital may have poorer control of diabetes or have other risk factors for DKD such as hypertension and dyslipidemia, as compared to the patients who visit a practitioner or a community hospital. Therefore, our study may be biased with respect to the cohort at a high risk of developing DKD. Additionally, data from a single-center cannot be representative nor generalized for general clinic and hospitals. Secondly, proteinuria and albuminuria were not measured quantitatively as compared to the estimation of microalbuminuria in previous large-scale analyses [36,37]. We regarded proteinuria 1+ with dipstick urine test as equivalent to 30–300 mg/gCr since the majority of proteinuria 1+ and higher (80–98%) have been reported to be albuminuria positive [38,39]. Thirdly, hypertension is an important risk factor for DKD, but hypertension was not associated with DKD. The current study might be biased for hypertension for the following reasons: the prevalence of hypertension was high both in DD (75.0%) and UD (68.9%) groups, and blood pressure was controlled during follow-up extensively by specialists for diabetes and hypertension.

## 5. Conclusions

The results of our study showed for the first time that UD is a strong risk factor for developing DKD. The notion that a longer duration of untreated diabetes mellitus is a risk factor for developing DKD warranted us to identify and scrutinize UD patients carefully.

## Figures and Tables

**Table 1:** Baseline characteristics of study participants.

	Diagnosed	Undiagnosed	*p* Value
*n*	164	244	
Age, years	56.3 ± 11.3	56.4 ± 11.1	0.946
Female,%	47.0	43.9	0.537
BMI, kg/m^2^	25.5 ± 4.9	25.4 ± 5.7	0.847
Current or past smoking,%	57.3	48.0	0.063
Family history of diabetes,%	39.0	47.5	0.089
Systolic blood pressure, mmHg	135 ± 20.6	136 ± 19.8	0.605
Diastolic blood pressure, mmHg	81.6 ± 13.1	79.5 ± 12.9	0.113
Hypertension,%	75.0	68.9	0.178
Dyslipidemia,%	74.4	70.5	0.390
Retinopathy,%	6.7	20.5	<0.001
Coronary artery disease,%	18.3	16.8	0.697
Stroke,%	8.5	9.8	0.658
Plasma glucose, mg/dL	150 (119–208)	167 (129–238)	0.006
HbA1c,%	7.1 ± 2.1	8.3 ± 2.1	<0.001
HbA1c, mmol/mol	54	67	<0.001
LDL cholesterol, mg/dL	117 ± 37.1	118 ± 36.0	0.941
HDL cholesterol, mg/dL	50.9 ± 13.7	50.0 ± 15.1	0.523
Triglycerides, mg/dL	131 (91.0–185)	118 (89.0–173)	0.311
eGFR, mL/min/1.73 m^2^	89.1 ± 22.7	88.9 ± 20.6	0.942

Data are presented as means ± SD, median (25–75th percentile), or percentages. *p* values are obtained by Chi-square test, Student′s t-test, and Mann–Whitney *U* test between diagnosed and undiagnosed diabetes groups. HhubA1c: Hemoglobin A1c; eGFR: estimated glomerular filtration rate.

**Table 2:** Univariate and multivariate hazard ratio for onset of DKD.

				Multivariate Analysis								
	Univariate Analysis			Model 1			Model 2			Model 3		
Variables	Crude HR	95% CI	*p* Value	Adjusted HR	95% CI	*p* Value	Adjusted HR	95% CI	*p* Value	Adjusted HR	95% CI	*p* Value
Undiagnosed diabetes mellitus, yes or no	1.760	(1.323–2.341)	<0.001	1.706	(1.271-2.290)	<0.001	1.647	(1.223–2.219)	0.001	1.566	(1.159–2.115)	0.003
Age, per year	1.008	(0.995–1.021)	0.221	1.010	(0.995–1.024)	0.201	1.007	(0.992–1.022)	0.385	1.009	(0.994–1.024)	0.233
Male gender, yes or no	1.471	(1.121–1.932)	0.005	1.451	(1.094–1.926)	0.010	1.500	(1.065–2.113)	0.020	1.439	(1.020–2.031)	0.038
BMI, kg/m^2^	1.001	(0.977–1.026)	0.926	1.014	(0.986–1.042)	0.331	1.010	(0.983–1.038)	0.466	1.012	(0.985–1.039)	0.399
Baseline HbA1c,%	1.039	(0.980–1.101)	0.204	1.029	(0.964–1.099)	0.387	1.021	(0.951–1.097)	0.564	0.977	(0.900–1.060)	0.574
Baseline eGFR, mL/min/1.73 m^2^	0.993	(0.986–1.000)	0.042	0.995	(0.988–1.003)	0.227	0.966	(0.988–1.003)	0.258	0.994	(0.986–1.002)	0.118
Current or past smoking, yes or no	1.101	(0.843–1.436)	0.480				0.972	(0.701–1.348)	0.864	0.986	(0.710–1.370)	0.935
Hypertension, yes or no	1.231	(0.910–1.665)	0.178				1.257	(0.909–1.737)	0.167	1.175	(0.850–1.624)	0.329
Dyslipidemia, yes or no	0.834	(0.625–1.113)	0.218				0.878	(0.651–1.184)	0.394	0.845	(0.626–1.141)	0.271
Macroangiopathy, yes or no	0.974	(0.714–1.328)	0.867				0.910	(0.656–1.263)	0.573	0.936	(0.674–1.300)	0.694
Retinopathy, yes or no	1.519	(1.077–2.141)	0.017				1.415	(0.970–2.064)	0.072	1.287	(0.877–1.888)	0.197
Median HbA1cat follow-up,%	1.213	(1.099–1.338)	<0.001							1.244	(1.086–1.424)	0.002
